# Ultra‐mutated colorectal cancer patients with *POLE* driver mutations exhibit distinct clinical patterns

**DOI:** 10.1002/cam4.3579

**Published:** 2020-10-30

**Authors:** Hanguang Hu, Wen Cai, Dehao Wu, Wangxiong Hu, Li Dong Wang, Jianshan Mao, Shu Zheng, Weiting Ge

**Affiliations:** ^1^ Department of Medical Oncology The Second Affiliated Hospital School of Medicine Zhejiang University Hangzhou Zhejiang China; ^2^ Cancer Institute (Key Laboratory of Cancer Prevention and Intervention, China National Ministry of Education) The Second Affiliated Hospital School of Medicine Zhejiang University Hangzhou Zhejiang China; ^3^ Department of Gastroenterology Second Affiliated Hospital of Zhejiang University School of Medicine Hangzhou China; ^4^ Henan Key Laboratory for Esophageal Cancer Research of the First Affiliated Hospital State Key Laboratory for Esophageal Cancer Prevention & Treatment Zhengzhou University Zhengzhou Henan China

**Keywords:** clinical patterns, colorectal cancer, driver mutation, heterogeneity, POLE

## Abstract

*POLE* mutations, which lead to an ultramutated phenotype in colorectal cancer (CRC), have been reported as a promising marker in immunotherapy. We performed sequencing of CRC cases in Zhejiang University (ZJU) and extracted obtainable data from recently published results, including The Cancer Genome Atlas (TCGA), Japanese studies and clinical trials, to present clinical patterns of *POLE* driver‐mutated CRC and reveal its heterogeneity. The rate of somatic *POLE* driver mutations has been reported as 2.60% (ZJU cohort), 1.50% (TCGA cohort), 1.00% (Japan cohort), and 1.00% (Lancet cohort). *POLE* driver mutations show a clearly increased mutation burden (mean TMB: 217.98 mut/Mb in ZJU; 203.13 mut/Mb in TCGA). Based on pooled data, more than 70.00% of patients with *POLE* driver mutations were diagnosed before they were 55 years old and at an early disease stage (Stage 0–II >70.00%), and more than 70.00% were male. Among Asian patients, 68.40% developed *POLE* driver mutations in the left‐side colon, whereas 64.00% of non‐Asian patients developed them in the right‐side colon (*p* < 0.01). The top three amino acid changes due to *POLE* driver mutations are P286R, V411L, and S459F. Investigators and physicians should ascertain the heterogeneity and clinical patterns of *POLE* driver mutations to be better equipped to design clinical trials and analyze the data.

## Graphical abstract

*POLE* could be a promising marker for immunotherapy, and its heterogeneity should not be ignored. Here we present the clinical patterns and heterogeneity of POLE driver mutations so that investigators and physicians will be better equipped to design clinical trials and analyze their data.

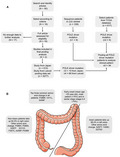

## 1 INTRODUCTION

DNA polymerase epsilon (*POLE*) is essential for proofreading in DNA replication, and mutations to *POLE* lead to DNA‐repair deficiencies.^1^ *POLE*‐mutated tumors have an extremely high tumor mutation burden (TMB), and the disruption by pathogenic heterozygous mutations found in cancers leads to a phenotype of tumor ultramutation, which produces an increase in neoantigens.^2^, ^3^

It is well known that patients with higher neoantigen levels may benefit from immunotherapy. Immunotherapy shows impressive efficacy in advanced cancer, but the exact target patient groups are still unclear, especially in CRC. Microsatellite instability (MSI‐high) is the only generally recognized marker for immunotherapy in CRC, and it only benefits 5.00% of CRC patients.^4^, ^5^ Therefore, more predictive markers are needed. *POLE* proofreading (exonuclease)‐domain mutations have been reported as promising candidate biomarkers.^6^, ^7^

Research on infrequent candidate prognostic biomarkers may be restricted by limitations of statistical power; nonetheless, many studies have reported that *POLE* mutations identify a subset of immunogenic CRCs with a better prognosis. Regardless, no studies have focused on the clinical pattern of *POLE* mutations, and limited data are available to characterize Asian patients or examine potential differences between racial groups, all of which are important for providing precision cancer medicine.

In this study, we performed sequencing in a Chinese cohort to conduct comprehensive genetic profiling of primary CRC. In addition, we extracted obtainable data from recently published results, including in TCGA, Japanese studies and clinical trials, to present clinical patterns of *POLE*‐mutated CRC and reveal the related heterogeneity with regard to race and primary sites in a large group of patients. These findings provide an important reference for future clinical trial design and data analysis.

## 2 MATERIALS AND METHODS

### 2.1 Literature search strategy

All related studies published were systematically searched using PubMed without language restrictions. The search strategies included the following key words: “*POLE*,” “DNA polymerases Pol ε” AND “colon” OR “rectum” OR “colorectal.” Two authors initially checked the titles and abstracts and obtained the full texts. Then, one author selected publications related to CRC. References from the relevant published articles were manually searched to identify all possible studies. Conference abstracts or case reports were excluded due to lack of sufficient data for further analysis.

### 2.2 Inclusion and exclusion criteria

Studies were eligible when they met the following initial inclusion criteria: (a) *POLE* mutations were detected by sequencing; (b) *POLE* mutations were somatic, not germline mutations; (c) sufficient data were provided to estimate the analysis. Studies were excluded if they (a) were case reports, conference abstracts or letters; (b) contained unpublished data; or (c) lacked sufficient data for the pooling analysis.

### 2.3 Article data extraction

Eligible articles were initially reviewed by two reviewers independently (W.C. and D.W.). Differences of opinion were resolved by consensus and consultation with a third investigator (W.G.). Data were then extracted independently by two authors (W.C. and D.W.) following a previous protocol. The following required information was collected: number of patients; tumor type; sample source; basic information of included patients (age, sex, race, TNM stage, and tumor location); and methods of detecting the *POLE* mutation.

### 2.4 Sample collection and genomic DNA preparation in our hospital

Tumor and matched normal mucosa‐derived DNA samples were purified using a QIAamp DNA mini kit (QIAGEN, Germany). Fresh tissue specimens were flash frozen in liquid nitrogen and stored at −80°C until genomic DNA isolation after surgery. The pathologic diagnosis was confirmed by reviewing hematoxylin and eosin (H&E)‐stained slides, and the samples were excluded if the tumor cells were <40.00%.^8^ All patient samples were diagnosed with primary CRC without chemotherapy prior to surgery. The stage was assessed using the 7th version of the American Joint Commission on Cancer guidelines. This study was conducted in accordance with the Declaration of Helsinki. All procedures were approved by the Institutional Review Board (IRB) of the Second Affiliated Hospital, School of Medicine of Zhejiang University under protocol 2013‐042.

### 2.5 DNA sequencing and detection of somatic mutations and indels

Whole‐genome sequencing, exome sequencing and target captured sequencing were performed using the Illumina platform (Novogene, China). A previously custom‐designed panel of 524 genes was occupied.^8^ The mutations in the target captured regions were subjected to further analysis. A mutation was defined as any nonsynonymous SNV or indel, including nonsense mutations, missense mutations, frameshift insertions and deletions, in‐frame insertions and deletions, and splice‐site mutations, among others. To normalize the tumor mutation burden (TMB) between panel sequencing and exome sequencing, the total number of mutations was divided by the coding region captured in each panel, which covered 2.889 megabases (Mb) in a 524‐gene panel as previously described.^8^ The somatic mutation dataset and the clinicopathological information for the cohort from TCGA were obtained from the project data portal TCGA (http://www.cbioportal.org) on October 9, 2019. We identified *POLE* driver mutations, a hypermutation phenotype (>10 mut/Mb) and an ultramutation phenotype (>100 mut/Mb) as previously reported (Figure S1).^9^

### 2.6 Statistical analyses

**FIGURE 1:**
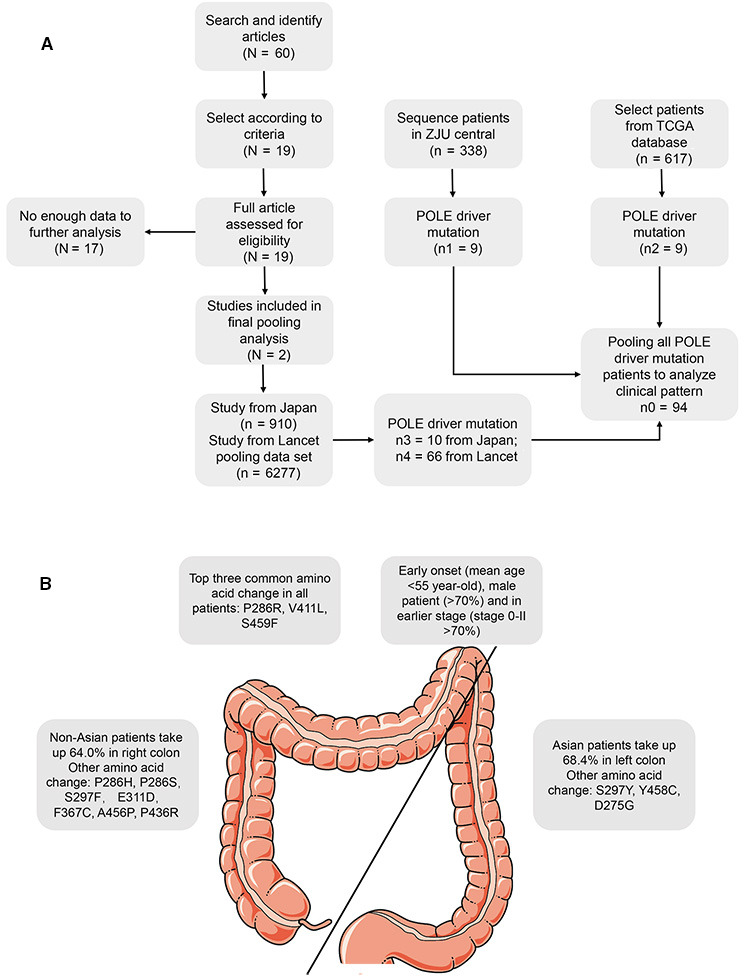
Flow diagram of the selection process and clinical pattern of CRC patients with POLE mutations

The patients’ demographic data and tumor characteristics were summarized using descriptive statistics. Univariate analyses were performed using the *χ* ^2^ test or Student's *t* test. All statistical analyses were performed using Intercooled Stata 12.0 (Stata Corporation, College Station, TX, USA). The results were considered statistically significant at a two‐sided *p* < 0.05. We present a selection flow chart in Figure 1.

## 3 RESULTS

### 3.1 Differences in *POLE* driver mutations between patients from the ZJU central and TCGA databases

We sequenced 338 CRC cases collected from the ZJU central database and analyzed 617 CRC patients from TCGA. Clinical information on the two cohorts is presented in Table 1. We extracted all cases with the hypermutated phenotype and divided them according to whether a *POLE* driver mutation was present. The *POLE* driver mutation group showed an obviously higher mutation burden (TMB) in the ZJU cohort (mean TMB: 217.98 mut/Mb vs. 77.53 mut/Mb), which was identified as an ultramutated phenotype. A similar difference was observed in the cohort from TCGA (mean TMB: 203.13 mut/Mb vs. 46.72 mut/Mb), although it is not appropriate to directly compare TMB values between two cohorts. Patients were diagnosed at early stages in both groups (Stage I and Stage II), but the primary sites were different. In the ZJU cohort, which only included Asian patients, *POLE* driver mutations were more likely to occur in the left‐side colon (left vs. right: 77.78% vs. 11.11%). In the cohort from TCGA, in which no Asian patients were included, *POLE* driver mutations were more likely to occur in the right‐side colon (left vs. right: 11.11% vs. 44.44%). The non‐*POLE* driver mutation patients in both cohorts were more likely to have primary sites in the right‐side colon. *POLE* driver mutations were present in 2.60% of the ZJU cohort and 1.50% of the cohort from TCGA. Patients with *POLE* driver mutations were younger than those in the non‐*POLE* driver mutation group (mean age: 54.89 years vs. 61.28 in ZJU; 55.00 years vs. 68.63 in TCGA).

**TABLE 1:** Characteristics of patients from ZJU and TCGA

	ZJU			TCGA		
	Hypermutation			Hypermutation		
	POLE driver mutation	Non‐POLE driver mutation	All patients	POLE driver mutation	Non‐POLE driver mutation	All patients
Total	9	36	338	9	76	617
Age (Mean[Min–Max])	54.89 (43.62–66.16)	61.28(56.61–65.94)	62.62(61.34–63.90)	55(43.89–66.11)	68.63(65.31–71.96)	66.43(65.42–67.44)
Male, n (%)	6(66.67)	23(63.89)	225(66.57)	6(66.67)	34(44.74)	326(52.84)
Asian, n (%)	9(100.00)	36(100.00)	338(100.00)	0(0.00)	0(0.00)	0(0.00)
Family history, n (%)	3(33.33)	4(11.11)	52(15.38)	0(0.00)	7(19.40)	73(13.62)
MSI‐H, n (%)	2(22.22)	20(55.56)	22(6.51)	0(0.00)	10(15.62)	46(12.92)
Muts/Mb(Mean[Min–Max]^a^	217.98(163.66–272.31)	77.53(30.68–44.38)	13.20(9.17–17.24)	203.13(129.05–277.21)	46.72(40.77–52.67)	12.85(10.08–15.62)
TNM stage, n (%)						
Stage I	0(0.00)	4(11.11)	35(10.36)	2(20.00)	3(8.30)	109(18.08)
Stage II	7 (77.78)	20(55.56)	135(39.94)	7(70.00)	25(69.40)	220(36.48)
Stage III	2(22.22)	11(30.56)	123(36.39)	1(10.00)	5(13.90)	182(30.18)
Stage IV	0(0.00)	1(2.78)	45(13.31)	0(0.00)	1(2.80)	92(15.26)
Primary site						
Right‐sided Colon, n (%)	1(11.11)	21(58.33)	94(27.81)	4(44.44)	61(84.72)	261(43.57)
Left‐sided Colon, n (%)	7(77.78)	7 (19.44)	87(25.74)	1(11.11)	5(6.94)	175(29.22)
Rectum, n (%)	1(11.11)	8(22.22)	157(46.45)	4(44.44)	6(8.33)	163(27.21)

^a^ Muts/Mb: mutations per megabase. To normalized tumor mutation burden between panel sequencing (ZJU) and exome sequencing (TCGA), the total number of mutations was divided by the coding region captured in each panel, which coved 2.889 Mb in a 524‐gene panel.

### 3.2 Patients collected from published articles

We systematically searched articles published in the database and retrieved two articles that met our criteria. We extracted the data from these two articles, as presented in Table 2. Data from the Japanese research,^10^ which included 910 CRC patients, only included patients from Japan; the article from the Lancet pooling data from clinical trials included 6277 non‐Asian CRC patients.^11^ We can conclude from the pooled the data that patients with *POLE* driver mutations are younger than 55 years old and are diagnosed at earlier stages (Japanese research vs. Lancet pooling research: 80.00% vs. 69.70%). The primary sites for patients with *POLE* driver mutations were also different in the two cohorts, although there were more patients with left‐side colon cancer in both cohorts (Japanese research vs. Lancet pooling research: 68.10% vs. 57.40%). In the Japanese cohort, which only included Asian patients, *POLE* driver mutations were more likely to occur in the left‐side colon (left vs. right: 60.00% vs. 40.00%). In the Lancet pooled sample, in which no Asian patients were included, POLE driver mutations were more likely to occur in the right‐side colon (left vs. right: 30.30% vs. 66.70%).

**TABLE 2:** Characteristics of patients from articles

	POLE driver mutation			All patients		
	Japanese research	Lancet pooling research	*p*‐value	Japanese research	Lancet pooling research	*p*‐value
Total	10	66		910	6277	
Age (Mean[Min–Max])	43(30–85)	54(27–82)		67(20–93)	68(19–100)	
Asian, n(%)	10(100.00)	0(0.00)		910(100.00)	0(0.00)	
Male, n (%)	9(90.00)	50(75.80)	0.55	532(58.50)	3496(55.70)	0.12
MSI‐H, n (%)	unreported	0(0.00)		unreported	833(100)	
TNM stage, n (%)			0.88			0.93
Stage 0–II	8(80.00)	46(69.70)		438(48.10)	2996(47.70)	
Stage III–IV	2(20.00)	18(27.30)		472(51.90)	3249(51.80)	
Primary site			0.08			<0.01
Right‐sided Colon, n (%)	4(40.00)	44(66.70)		290(31.90)	2381(37.90)	
Left‐sided Colon, n (%)	6(60.00)	20(30.30)		620(68.10)	3603(57.40)	

### 3.3 Pooling data to present the characteristics of *POLE* driver mutations

To investigate the characteristics of patients with *POLE* driver mutations, we further pooled data from ZJU, TCGA and the Japanese and Lancet research. The results are presented in Table 3. The final pooled data suggested that *POLE* driver mutations occurred more frequently in male patients (Asian vs. Non‐Asian: 78.90% vs. 74.60%; *p* = 1.00) and at early stages (Stage 0–II) of CRC (Asian vs. Non‐Asian: 78.90% vs. 73.30%; *p* = 0.73). Detection of *POLE* driver mutations in different primary sites varied by race (*p* < 0.01). In Asian patients, *POLE* driver mutations occurred more frequently in the left‐side colon (right vs. left: 26.30% vs. 68.40%); in non‐Asian patients, they occurred more frequently in the right‐side colon (right vs. left: 64.00% vs. 28.00%).

**TABLE 3:** The clinical patterns of patients with POLE driver mutations

	Asian	Non‐Asian	*p*‐value
Total patients	19	75	
Age (Mean)	49.00	54.50	NA
Male, n (%)	15(78.90)	56(74.60)	1.00
TNM stage, n (%)			0.73
Stage 0–II	15(78.90)	55(73.30)	
Stage III–IV	4(10.50)	19(25.30)	
Primary site			<0.01
Right‐sided Colon, n (%)	5(26.30)	48(64.00)	
Left‐sided Colon, n (%)	13(68.40)	21(28.00)	
POLE driver mutation (Amino acid change)			<0.01
P286R	12(63.16)	37(49.33)	
V411L	2(10.52)	17(22.67)	
S459F	1(5.26)	10(13.33)	
A456P	0(0.00)	4(5.33)	
P436R	0(0.00)	2(2.67)	
S297Y	1(5.26)	0(0.00)	
Y458C	1(5.26)	0(0.00)	
D275G	1(5.26)	0(0.00)	
P286H	0(0.00)	1(1.33)	
P286S	0(0.00)	1(1.33)	
S297F	0(0.00)	1(1.33)	
E311D	0(0.00)	1(1.33)	
F367C	0(0.00)	1(1.33)	

### 3.4 Typical characteristics of *POLE* driver mutations and commonly mutated domains

According to the results of the pooled data, we summarized the characteristics of *POLE* driver mutations in Figure 1. All included patients with *POLE* driver mutations were relatively young (mean age <55 years old) and at an early stage of colon cancer (Stage 0–II >70.00%). More than 70.00% of patients with *POLE* driver mutations were male. The most common amino acid changes in association with *POLE* driver mutations across all patients were P286R, V411L, and S459F. There were significant differences between Asian and non‐Asian patients with regard to the development of *POLE* driver mutations, with 68.40% of Asian patients developing *POLE* driver mutations in the left‐side colon while 64.00% of non‐Asian patients developed them in the right‐side colon. The amino acid change also varied by location and race, but there were too few patients with rare amino acid changes to draw a firm conclusion.

## 4 DISCUSSION

Patients with *POLE* mutations who benefit from immunotherapy have recently been reported, which indicates that the *POLE* gene could be a potential marker for treating CRC patients; however, its clinical characteristics and potential heterogeneity should not be overlooked.^11^, ^12^, ^13^ In this study, we performed the genetic profiling of primary CRCs in patients from our central database. We also extracted obtainable data from recently published data, including TCGA, Japanese research and clinical trials, to investigate clinical patterns of *POLE*‐mutated CRC and reveal the potential heterogeneity in *POLE* mutations by race and primary site. To the best of our knowledge, this is the first study to compare the different clinical patterns of *POLE* driver mutations according to race and primary site.

Different sequencing panels and calculation methods may relate to significant differences of TMB. Therefore, calculation of TMB in two cohorts separately and division of patients into non‐hypermutation, hypermutation, and ultra‐mutation are reasonable. As shown in Figure S1, the threshold for the TMB groups was visually confirmed by an uptick in the slope of the line. POLE driver mutations presented an ultramutated phenotype (mean TMB >100 mut/Mb in the ZJU and TCGA cohorts), which was consistent with a previous report. We did not observe a distinct genetic predisposition in somatic *POLE* mutations. Previously, germline mutations in *POLE* and *POLD1* have been reported in patients with polymerase proofreading‐associated polyposis, implying that germline *POLE* and *POLD1* mutations are involved in familial CRCs.^14^, ^15^ However, there are still no studies confirming whether somatic *POLE* and *POLD1* mutations act as key drivers of CRC or revealing the related underlying pathway.^16^, ^17^

This research presents distinct clinical characteristics of patients with *POLE* driver mutations. All included patients with *POLE* driver mutations were younger than 55 years and diagnosed at an early stage (Stage 0 to II). In the pooled analysis, more than 70.00% of patients with *POLE* driver mutations were male. We also observed differences in age between Asian and non‐Asian patients. The above results indicate that these clinical characteristics should be adjusted in further multivariate analyses.

According to the data on amino acid changes underlying *POLE* driver mutations, we summarized the top three common amino acid changes: P286R, V411L, and S459F. Other changes, such as Q125H and R259H, were not associated with an ultramutated phenotype.

We are the first group to report that *POLE* driver mutations are present at primary sites that significantly differ between Asian and non‐Asian patients. We observed *POLE* driver mutations in the left‐side colon in 68.40% of Asian patients, whereas 64.00% of non‐Asian patients developed them in the right‐side colon. Additionally, the amino acid changes underlying *POLE* mutations differed between the left‐ and right‐side colon. Currently, increasing research is focused on finding new markers for immunotherapy, and *POLE* gene mutations are considered a promising marker in various cancer types.^18^, ^19^, ^20^ We advocate that more clinical trials should be focused on detecting *POLE* gene mutations to identify additional clinical features. Here, we present an overview of the clinical patterns of patients with *POLE* mutations and report significant differences among diverse groups, which could contribute to identifying specific groups of patients who could benefit from immunotherapy.

CRC patients with *POLE* driver mutations present a distinct clinical pattern, including an ultramutated phenotype, a young age (less than 55 years old), a greater proportion of males than females and diagnosis at an early stage. *POLE* driver mutations present in significantly different primary sites between Asian and non‐Asian patients, with Asian patients commonly developing them in the left‐side colon and non‐Asian patients in the right‐side colon. Investigators and physicians should be aware of the potential heterogeneity and clinical pattern of *POLE* driver mutations to be better equipped to design clinical trials and analyses pertaining to the treatment of CRC.

## CONFLICT OF INTEREST

The authors have no conflict of interest.

5

## Supporting information

Supplementary Material

## Data Availability

Whole genome and exome capture sequencing data were deposited in the European Nucleotide Archive under study accession number EGAS00001001269. The somatic mutation dataset and the clinicopathological information of the TCGA cohort were obtained from the TCGA project data portal (http://www.cbioportal.org).
