# Supplementary material for: Ultra‐mutated colorectal cancer patients with POLE driver mutations exhibit distinct clinical patterns
Source: Cancer Med. 2020 Oct 30;10(1):135–42. doi: 10.1002/cam4.3579 (PMC7826451; doi:10.1002/cam4.3579)
Supplement: Supplementary file 1 — Supplementary Material [file CAM4-10-135-s001.docx]

**Supplementary Figure 1. The thresholds of non-hypermutation, hypermutation and ultramutation in ZJU and TCGA cohorts.**

a


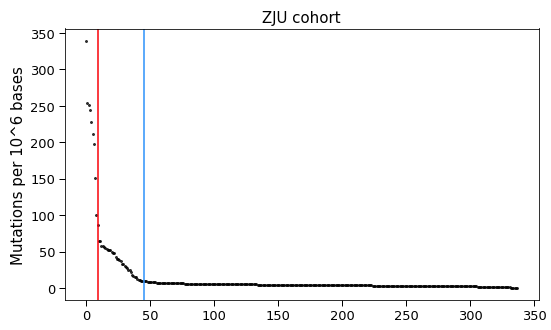


b


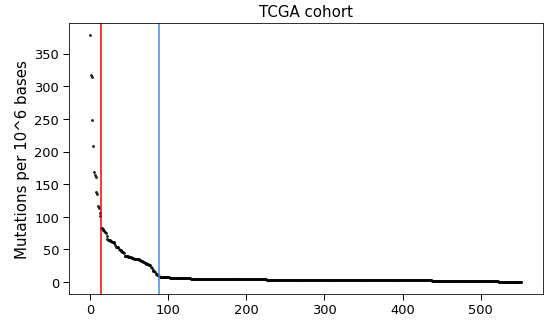


Red line indicates a threshold of 100 mutations/Mb and blue line indicates a threshold of 10 mutations/Mb.

**Supplementary Table 1: Mutation frequency of vital CRC-related genes in the ZJU and TCGA cohorts.**

|  | Primary site | ZJU  (Asian) | TCGA  (non-Asian) | *P-*value |
| --- | --- | --- | --- | --- |
| Total patients | Right-side colon | 94 | 261 |  |
|  | Left-side colon | 87 | 175 |  |
|  | Rectum | 157 | 163 |  |
| APC mutated | Right-side colon | 50 (53.2%) | 158 (60.5%) | 0.264 |
|  | Left-side colon | 59 (67.8%) | 121 (69.1%) | 0.939 |
|  | Rectum | 118 (75.2%) | 115 (70.6%) | 0.424 |
| TP53 mutated | Right-side colon | 46 (48.9%) | 112 (42.9%) | 0.375 |
|  | Left-side colon | 58 (66.7%) | 103 (58.9%) | 0.276 |
|  | Rectum | 102 (65.0%) | 103 (63.2%) | 0.829 |
| KRAS (12/13  codon) | Right-side colon | 32 (34.0%) | 100 (38.3%) | 0.542 |
|  | Left-side colon | 15 (17.2%) | 37 (21.1%) | 0.561 |
|  | Rectum | 43 (27.4%) | 48 (29.4%) | 0.776 |
| BRAF V600E | Right-side colon | 9 (9.6%) | 42 (16.1%) | 0.169 |
|  | Left-side colon | 1 (1.1%) | 2 (1.1%) | 1 |
|  | Rectum | 0 (0%) | 0 (0%) | NA |

NA: not applicable.

**Supplementary Table 2:** Three-year survival rate of patients from the pooled data for ZJU and TCGA.

| Primary site | POLE driver mutation | Patient number | Three-year survival rate |
| --- | --- | --- | --- |
| Left-side colon | Yes | 7 | 85.7% |
|  | No | 146 | 75.3% |
| Right-side colon | Yes | 4 | 75.0% |
|  | No | 195 | 61.5% |
